# Influence of physician networks on the implementation of pharmaceutical alternatives to a toxic drug supply in British Columbia

**DOI:** 10.1186/s13012-023-01331-x

**Published:** 2024-01-06

**Authors:** Megan Kurz, Brenda Carolina Guerra-Alejos, Jeong Eun Min, Brittany Barker, Bernadette Pauly, Karen Urbanoski, Bohdan Nosyk

**Affiliations:** 1 Centre for Advancing Health Outcomes, Vancouver, BC Canada; 2 Faculty of Health Sciences, Simon Fraser University, Burnaby, V5A 1S6 Canada; 3 First Nations Health Authority, Vancouver, BC Canada; 4 School of Public Health and Social Policy, University of Victoria, Victoria, BC Canada; 5 Department of Nursing, University of Victoria, Victoria, BC Canada; 6 Canadian Institute for Substance Use Research, University of Victoria, Victoria, BC Canada

**Keywords:** Prescribed safer supply, Substance use disorder, Social network analysis, Implementation science

## Abstract

**Background:**

Characterizing the diffusion of adopted changes in policy and clinical practice can inform enhanced implementation strategies to ensure prompt uptake in public health emergencies and other rapidly evolving disease areas. A novel guidance document was introduced at the onset of the COVID-19 pandemic in British Columbia (BC), Canada, which supported clinicians to prescribe opioids, stimulants, and benzodiazepines. We aimed to determine the extent to which uptake and discontinuation of an initial attempt at a prescribed safer supply (PSS) program were influenced through networks of prescribers.

**Methods:**

We executed a retrospective population-based study using linked health administrative data that captured all clinicians who prescribed to at least one client with a substance use disorder from March 27, 2020, to August 31, 2021. Our main exposure was the prescribing patterns of an individuals’ peers, defined as the proportion of a prescribers’ professional network (based on shared clients), which had previously prescribed PSS, updated monthly. The primary outcome measured whether a clinician had prescribed their initial PSS prescription during a given calendar month. The secondary outcome was the discontinuation of PSS prescribing, defined as an absence for PSS prescriptions for at least 3 months. We estimated logistic regression models using generalized estimated equations on monthly repeated measurements to determine and characterize the extent to which peer networks influenced the initiation and discontinuation of PSS prescribing, controlling for network, clinician, and caseload characteristics. Innovators were defined as individuals initiating PSS prior to May 2020, and early adopters were individuals initiating PSS after.

**Results:**

Among 14,137 prescribers treating clients with substance use disorder, there were 228 innovators of prescribed safer supply and 1062 early adopters through the end of study follow-up, but 653 (50.6%) were no longer prescribing by August 2021. Prescribers with over 20% of peers whom had adopted PSS had a nearly fourfold higher adjusted odds of PSS prescribing themselves (*aOR*: 3.79, 95% *CI*: (3.15, 4.56)), compared to those with no connected safer supply prescribers.

**Conclusions:**

The uptake of PSS in BC was highly dependent on the behavior of prescribers’ peer networks. Future implementation strategies to support PSS or other policies would benefit from leveraging networks of prescribers.

**Supplementary Information:**

The online version contains supplementary material available at 10.1186/s13012-023-01331-x.

Contributions to the literature

- This study provides quantitative estimations of the effect of patient-sharing networks on a prescribers’ uptake of a novel prescribing guidance for prescribed safer supply using population-level administrative databases during an ongoing public health emergency.
- The study showed that prescribers whose peers had previously prescribed under the new guidance were associated with a higher probability of uptake of prescribed safer supply.
- Network effects for uptake of a novel policy were as large as other prescriber-level effects such as substance use disorder client load and prescribing history.

## Introduction

On March 26, 2020, the provincial government in British Columbia (BC), Canada, released what was termed risk mitigation guidance [1] to support people who use substances, an initial attempt at prescribed safer supply (PSS). The guidance document was introduced in order to promote self-isolation and thus prevent the spread of COVID-19 among people who use substances, as well as offering a medicalized harm reduction response to the increasingly toxic unregulated drug supply [2]. Risk mitigation guidance permitted prescribers to prescribe opioids, stimulants, and benzodiazepines to individuals who were at high risk of or had a confirmed or suspected COVID-19 infection and/or high likelihood of substance-related and systemic harms [2]. While introduced as an interim emergency measure, it subsequently became a provincial policy in July 2021, for prescribers that were not a part of specific health authorities in the province and prescribed other safer supply options [3]. An update was released in January 2022 [4] reporting preliminary evidence for patient outcomes and diversion, highlighting that PSS was not a direct contributor to the rising rate of illicit drug toxicity deaths.

This guidance was disseminated through voluntary online educational materials and didactic lectures, thus constituting a “passive” dissemination strategy. Initially, the guidance was published as an online educational material when it was released, with voluntary webinars recorded and made available online on April 9, 2020, as well as the slide presentation used [5–7]. The guidance was subsequently updated in 2022 and disseminated similarly. Such strategies have been found to be insufficient for driving system-level change [8–10]. A 2008 review of systematic reviews suggested passive dissemination is not sufficient, and that at least one, but preferably multiple targeted implementation strategies, such as educational outreach, interactive educational interventions, or practical recommendations, should be employed to create awareness of new guidelines or policies [11]. A more recent review classified distributing educational material passively as having mixed effectiveness for increasing health professionals’ knowledge, attitudes, and willingness to follow guidelines, but being generally ineffective (less than one-third of studies demonstrated a positive effect), for process-related outcomes which included prescription of medications [12]. A mixed-methods study from Quebec found passive dissemination of practice recommendations was successful when delivered by an opinion leader or self-identified champion, if recipients had a high level of expertise, and when there were sufficient professional resources, such as retaining nurses and other clinical staff [13].

Implementation science focuses on active and planned efforts to mainstream an innovation [14–16]. Diffusion of innovation theory has been applied within implementation science to focus on the natural and passive spread of innovation through adopters and design strategies to engage target groups to adopt specific innovations [14, 16]. Healthcare provider networks play an important role in the process of implementing innovations in clinical practice [17]. Previous work has demonstrated patterns of influence among physicians in primary care practices [18]. Social network analysis (SNA) is increasingly used to provide evidence for interpersonal influences theorized in diffusion theory [19], by considering the connections among actors within a network to understand their patterns, influence, and relationships [20]. SNA can be used to pilot test the feasibility of an implementation [21]; design communication strategies [22] tailoring dissemination to opinion leaders, network isolates, and other prescribers with few clinical contacts; and evaluate implementation efforts [23]. Health administrative data can be used with SNA to model physicians’ social networks, creating opportunities to evaluate and inform the implementation and diffusion of new policies and interventions on a population level [24, 25]. Previous research on diffusion of new policies has shown the influence of networks on tobacco regulations among countries [26]. In cancer treatment, physicians connected to previous prescribers of bevacizumab had higher odds (adjusted odds ratio 1.64; 95% confidence interval, 1.20–2.25) of prescribing the following year [27], with other medications having similar effects [28]. These studies are based on well-established, and less contentious, treatment effectiveness than PSS. Therefore, PSS presents a unique opportunity to understand the spread of a new emergency policy that faced pushback from addiction medicine prescribers, and other specialties, thus challenging the influence exerted by peers.

Treatment specific to opioid use disorder such as methadone and buprenorphine-naloxone, the primary modalities of opioid agonist treatment (OAT), is available in both specialized treatment centers and office-based settings [29]. Relaxed restrictions on OAT prescribing have allowed family physicians, nurse practitioners, and registered nurses to start opioid agonist treatment for opioid use disorders without any required waivers [30, 31]. Given that the risk mitigation guidance was applicable to all prescribers caring for people who use substances, a large diversity of prescribers, operating in different settings with varying levels of experience, caseloads of clients who use substances connections to other prescribers caring for people who use substances were eligible to participate. Analyzing the diffusion of PSS uptake among providers can provide vital information on how to adjust strategies to ensure intervention reach and adoption are optimized. We aimed to characterize the diffusion of PSS adoption and determine the extent to which PSS uptake operated through established networks of prescribers.

## Methods

### Study population and data sources

We executed a retrospective population-based study based on a linkage of ten provincial health administrative database that captures all individuals accessing care for a substance use disorder (SUD) in BC, Canada. The primary data source used was PharmaNet, which captures drug dispensations, prescriber identifiers, and client identifiers for all individuals regardless of private or public insurance coverage status. Other databases included the discharge abstract database [32] (DAD; records of hospitalizations), medical services plan [33] (MSP; physician billing records), BC Vital Statistics [34] (capturing deaths and their underlying cause), BC Provincial Corrections [35] (records of entry into incarcerations and releases to community), Perinatal Care Database [36] capturing maternal/infant care and outcomes), National Ambulatory Care Reporting System database [37] (NACRS; capturing emergency department visits), BC Social Development and Poverty Reduction database [38] (capturing social assistance receipt), BCCDC COVID-19-positive cases database [39] (capturing positive COVID-19 cases), and Provincial Laboratory Information Solution COVID-19 database [40] (PLIS; capturing COVID-19 lab tests). Descriptions of each database are available in the supplementary appendix (Table A1). Risk mitigation guidance was released on March 26, 2020; our study period covered March 27, 2020, to August 31, 2021, the last date available in our linked administrative databases.

As PSS was available to be prescribed by any physicians or nurse practitioners that care for individuals with SUD, our study population included physicians and nurse practitioners that prescribed any medications to at least one client with a prior indication of SUD. Clients with SUD were identified through receipt of OAT (Table A2), and three physician or nurse practitioner visits or at least one acute care visit in which the diagnostic code associated indicated a SUD (Table A3). Pregnant people could also be identified as having an SUD through records of perinatal care. Prescriber-client attachment was defined as any medication dispensation record between the prescriber and the client.

### Prescriber network construction

SNA was implemented to create a network of connections between prescribers based on their shared clients. A network is defined as a set of “actors” (prescribers) with “relational ties” (shared clients) [20]. As networks were constructed at the prescriber level, a “connection” between two prescribers indicates they prescribed any medication to at least one shared client. Within a given network, connections were weighted to represent the total number of shared clients, such that the greater the weighted tie, the more clients were shared between prescribers.

### Outcome

The primary outcome was assessed on the prescriber level each calendar month during the study period, indicating the initial PSS dispensation attributed to them. Prescribers’ follow-up ended when they initiated PSS. The secondary outcome for prescribers who had at least one PSS dispensation was the discontinuation of PSS, defined as an absence for PSS dispensations for at least 3 months. This outcome was evaluated from the start of PSS prescribing until discontinuation or the end of study follow-up.

### Primary exposure

We created the network exposure as proportion of connections following previous literature [41, 42]. We characterized the proportion of connected prescribers that had provided PSS in the months prior by including all dispensations provided to clients that were classified as PSS via a case-finding algorithm (Tables A4–5). This case-finding algorithm was necessary as new drug identification numbers were not assigned for PSS medications. Two separate algorithms have been proposed that identify prescriptions based on key words in the “directions for use” which is specified by the prescriber [2]. We chose the more sensitive, less specific algorithm for this analysis but included a sensitivity analysis for the more specific classification. The network exposure was updated each calendar month to capture the change in a prescriber’s peers’ PSS uptake over the follow-up period. We used a cut-off threshold based on the 3rd quartile of the empirical distribution of the network exposure (i.e., > 20% of connected peers prescribed PSS) to allow flexible assumptions on the relationship as well as reduce the influence of extreme observations since uptake was limited, reflecting smaller proportions of connections prescribers.

### Control variables

We controlled for covariates measured at the network-level and prescriber-level and also adjusted for client case mix. Network-level covariates included a clustering coefficient, measuring how connected the clinician and their neighbors are through the proportion of neighboring prescribers that are also connected, and adjusted strength which we defined as the number of additional prescribers their mean clients are connected with. Clinician-level measures included the prescribers’ primary practice location (Interior, Fraser, Vancouver Coastal, Vancouver Island, or Northern Regional Health Authority), their specialty (general practitioner, nurse practitioner, psychiatrist, other specialty, or unknown specialty), history of OAT prescription, years of experience treating clients with SUD, and their SUD client load. Prescriber-level variables were categorized into quartiles from their respective empirical distributions. Client case-mix measures included clients’ average chronic disease score [43, 44], benzodiazepine prescriptions, receipt of social assistance, drug-related acute care visit history, and COVID-19 diagnosis (via a positive lab record or inclusion in the BCCDC-positive case database), all aggregated to the prescriber-level. All covariates were updated on a monthly basis. All historical data (dating back to January 1, 1996) was used to construct variables on client and prescriber characteristics (e.g., history of OAT and prescribing experience were calculated from the first record after 1996).

### Statistical analysis

We first summarized the individual characteristics of prescribers stratified by early, late, and non-adopter status at time of PSS uptake or end of follow up and presented the number of active PSS prescribers by each calendar month in the study follow-up. However, we do not include test statistics with *p*-values to test the differences groups as we are not interested in simply describing statistically significant differences. PSS innovators were defined as prescribers whose first PSS dispensation occurred in March or April 2020. Thus, any other prescribers whose first PSS dispensation was after April 2020 were defined as an early adopter. We use this definition to separate those that learned about PSS through educational and training sessions that were provided through the BC center on substance use in April 2020. We estimated logistic regression models using generalized estimating equations [45] with an unstructured correlation matrix on monthly repeated measurements to determine and characterize the extent to which peer networks influenced the initiation and discontinuation of PSS prescribing, controlling for prescriber-level characteristics. A generalized estimating equation model was chosen as we had found the random effect variance estimate to be equal to 0 in a generalized linear mixed model. The regression analysis included observations from May 2020 where covariates and connected prescribers were measured in previous month, starting in April 2020. The focus of the regression is to measure the influence of peer prescribing on individuals, so by starting in May 2020, the analysis would exclude early adopters that are not subjected to peer influence. Data sets were constructed and analyzed in SAS version 9.4 [46].

### Sensitivity analyses

To ensure robustness of our results and estimates, we performed several sensitivity analyses. We assessed the influence of degrees of separation up to 2° in the network (i.e., peers of peers) [47]. We then reclassified the exposure of peer influence to the proportion of weighted edges attached to a connected PSS prescriber, as higher weights imply more shared clients, which may suggest a stronger connection between clinicians. We also added an additional analysis starting in July 2020, to assess a longer “innovation” period. We also included a sensitivity analysis that assessed monthly measurements between the 16th of each month to the 15th of the next calendar month. To assess if physicians treating very few SUD clients skewed our findings as their adoption may be unlikely, we included two sensitivity analyses with a threshold on the prescriber’s clients load as an additional inclusion criterion (more than one client and at least five clients). Lastly, we executed subgroup analysis on the PSS uptake outcome to focus on specific PSS medications: opioids (hydromorphone tablets, sustained-release oral morphine), stimulants (dextroamphetamine or methylphenidate), or benzodiazepines (diazepam or clonazepam).

## Results

### Descriptive characteristics of prescribers

A total of 14,137 prescribers treated 165,025 clients with an indication of SUD between March 27, 2020, and August 31, 2021, in British Columbia, Canada (Table 1), 228 (1.6%) of whom were PSS innovators (initial PSS prescription: March 27, 2020–April 30, 2020), 1062 (7.5%) early adopters (May 1, 2020–August 31, 2021), and 12,847 (90.9%) non-adopters. Innovators were largely practicing within the Vancouver Coastal Health Authority (138; 60.5%), whereas early and non-adopters were distributed more proportionally throughout the province. Prescribers practicing in the Fraser Health Authority were more commonly non-adopters (3178; 24.7% compared to 199; 18.7% early adopters). Innovators were primarily OAT prescribers (207; 90.8%), with a median of 73 shared clients (IQR bounds of 40 and 118). Early adopters had a median of 22 shared clients, while non-adopters only had a median of 4 shared clients. Innovators had cared for a median of 64 SUD clients (IQR bounds of 34 and 99), while early adopters saw a median of 21 clients (IQR bounds of 8 and 43), and non-adopters had a median of only 3 SUD clients (IQR bounds of 1 and 9). Innovators and early adopters had similar proportions of connected clinicians prescribing PSS. Non-adopters had a median of 21% of clients accessing social assistance, whereas early adopters had a median of 51.8%, and innovators had a median of 77.2%. A total of 63.4% (673) of early adopters were no longer prescribing by August 2021 (673; 63.4%), but only 27.2% (62) of innovators (62; 27.2%) stopped prescribing.

**Table 1:** Characteristics of prescribers by PSS adoption status at PSS initiation

	PSS innovators (*n* = 228)	Early PSS adopters (*n* = 1062)	PSS non-adopters^a^ (*n* = 12847)
**Practice characteristics**	N (%)		
Primary HA practice location			
*Interior*	14 (6.1%)	165 (15.5%)	1938 (15.1%)
*Fraser*	31 (13.6%)	199 (18.7%)	3178 (24.7%)
*Vancouver Coastal*	138 (60.5%)	387 (36.4%)	4392 (34.2%)
*Vancouver Island*	40 (17.5%)	215 (20.2%)	2258 (17.6%)
*Northern*	< 10^b^	86 (8.1%)	590 (4.6%)
*Missing (8 or 9)*	< 10^b^	10 (0.9%)	491 (3.8%)
Prescriber speciality			
*Nurse practitioner*	32 (14.4%)	88 (8.3%)	516 (4.0%)
*General practice*	175 (76.8%)	726 (68.4%)	7005 (54.5%)
*Psychiatry*	≤ 10^b^	109 (10.3%)	530 (4.1%)
*Known but other specialties*	≤ 10^b^	68 (6.4%)	3411 (26.6%)
*Unknown (physician)*	≤ 10^b^	71 (6.7%)	1385 (10.8%)
OAT prescriber	207 (90.8%)	799 (75.2%)	3838 (29.9%)
Discontinued as of end of follow-up	62 (27.2%)	673 (63.4%)	-------
**Network characteristics**	Median (1st quartile-3rd quartile)		
Number of connected physicians	29 (17–49)	16 (9–27)	3 (1–8)
Total sum of all shared clients	73 (40–118)	22 (11–47)	4 (1–9)
Proportion of connected physicians that prescribed PSS	0.34 (0.18–0.46)	0.38 (0.22–0.59)	0.13 (0–0.33)
Weighted proportion of PSS prescribers	0.50 (0.26–0.65)	0.44 (0.25–0.68)	0.11 (0–0.33)
Proportion of connected physicians that are also connected	0.15 (0.10–0.22)	0.19 (0.11–0.31)	0.14 (0–0.36)
Number of additional physicians their average clients saw	1.2 (0.9–1.7)	1.1 (0.8–1.8)	1.0 (0.5–1.5)
Degree centrality	0.20 (0.12–0.30)	0.08 (0.05–0.03)	0.02 (0.01–0.04)
**SUD prescribing history**			
Years since first billing record for a client with SUD	8.2 (4.3–17.2)	7.9 (3.1–18.2)	10.0 (3.7–21.7)
**SUD client caseload**			
SUD client load	63 (34–99)	21 (8–43)	3 (1–9)
Mean clients’ years since SUD diagnosis	12.2 (10.4–13.9)	11.8 (9.9–13.6)	12.0 (8.5–15.0)
Treated a client with a COVID-19 diagnosis	< 10^b^	79 (7.4%)	386 (3.0%)
Percent clients with a CCI of 1 or higher	8.7 (5.1–13.1)	7.7 (2.4–14.3)	0 (0–21.1)
Percent clients with a CDS over 4	1.3 (0–2.9)	0 (0–2.9)	0 (0–0)
Percentage of clients prescribed benzos	10.0 (5.6–15.9)	14.3 (6.5–24.2)	0 (0–20.4)
Percentage of clients accessing social assistance during past 12 months	77.2 (62.1–84.4)	51.8 (36.1–71.4)	20.8 (0–50.0)
Percentage of clients experienced an overdose 12 months prior	1.7 (0–3.8)	0 (0–3.0)	0 (0–0)

*Abbreviations*: *PSS* prescribed safer supply, *HA* health authority, *OAT* opioid agonist treatment, *SUD* non-opioid substance use disorder, *OUD* opioid use disorder, *CCI* Charlson comorbidity index

^a^Measured at end of follow-up (August 2021)

^b^Due to data sharing agreement, cell sizes < 10 are supressed

**Fig. 1:**
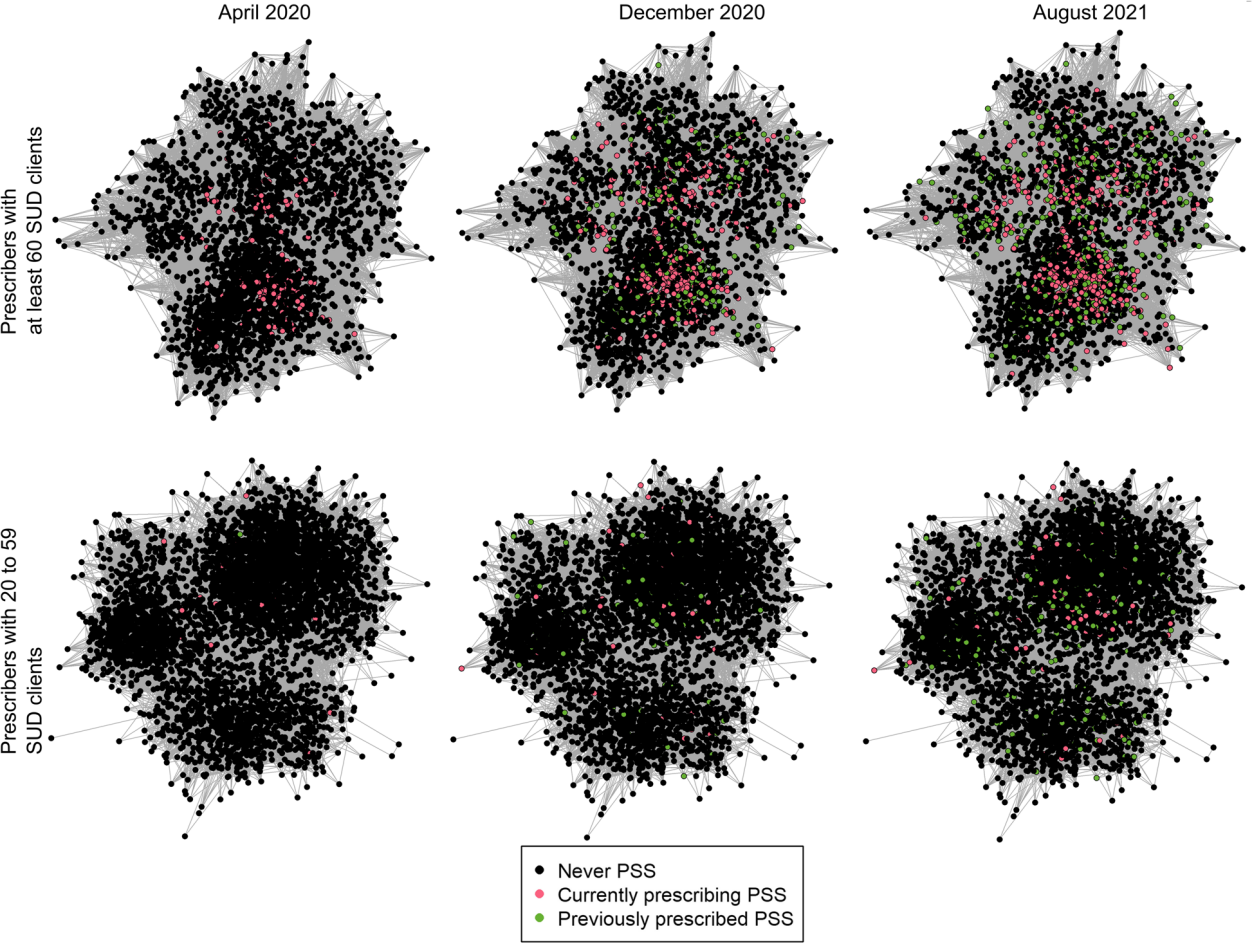
The networks of physicians and nurse practitioners that had at least 20 SUD clients between March 28, 2020, and August 31, 2021, colored by their PSS status at 3 separate months Abbreviations: SUD, substance use disorder; PSS, prescribed safer supply. A connection between two prescribers is kept if the weight (number of shared clients) was in the top 20% of connections for both prescribers. This requirement, along with SUD client load, was only considered for ease of display purposes. Each month displays the cumulative number of active PSS prescribers by the end of the month. Those that are currently prescribing PSS are displayed in pink. Those that prescribed PSS in previous months, but not the current month, are displayed in green. Non-PSS prescribers with SUD clients are displayed in black

**Fig. 2:**
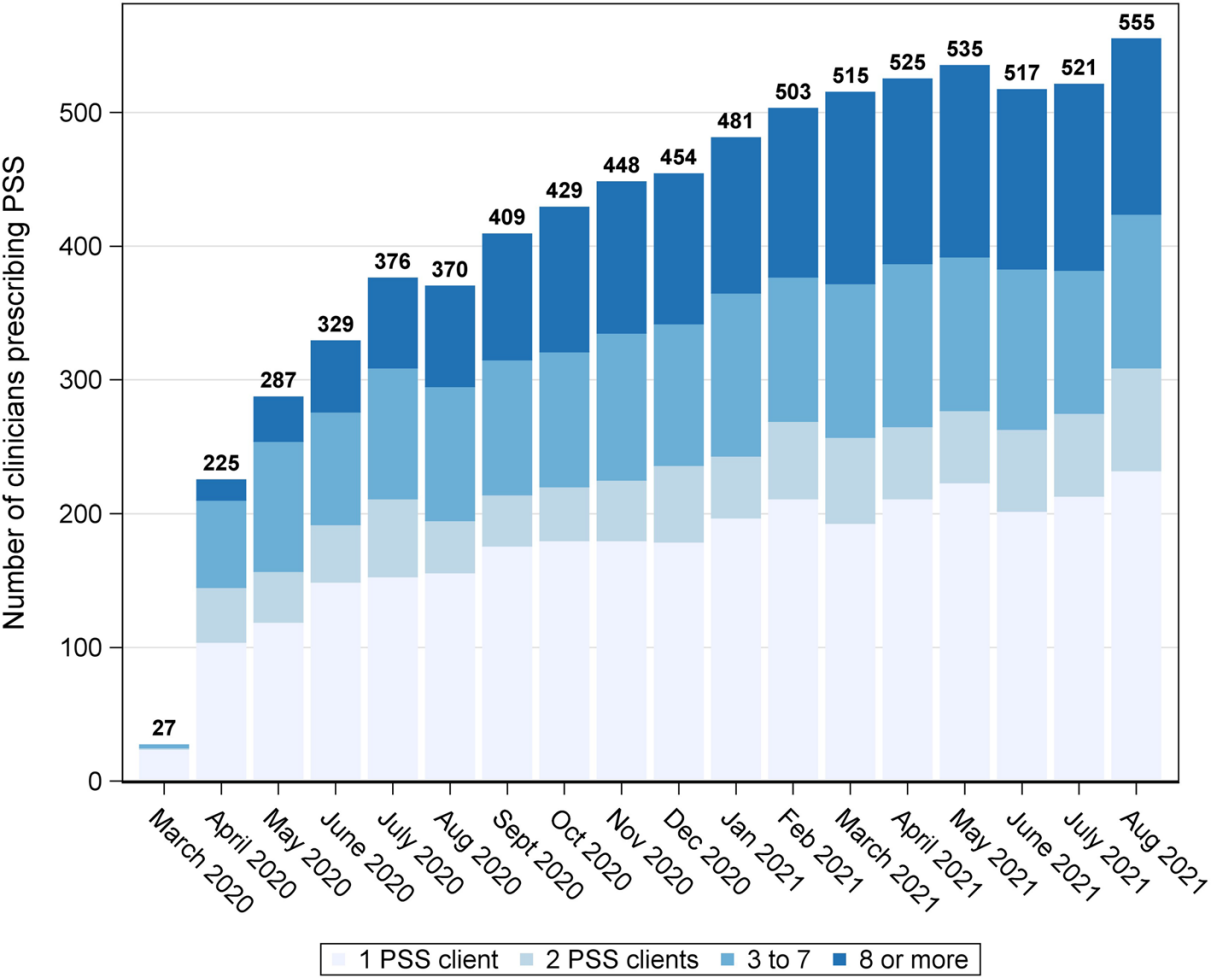
Summary of clinicians with at least one active prescribed safer supply (PSS) dispensation within the calendar month between March 2020 and August 2021

Prescriber initiation of PSS gradually increased over time, primarily through the largest clusters of prescribers (Fig. 1). However, there were only 555 active prescribers in August 2021; thus, 734 (56.9%) prescribers that were part of PSS uptake were no longer prescribing (Fig. 2).

### Multiple regression analysis on PSS adoption

A total of 153,869 prescriber months of follow-up were included in the analysis. SUD client load had the largest association with initiation of PSS (Table 2). Compared to having ≤ 2 SUD clients, having ≥ 14 SUD clients was associated with higher odds of prescribing PSS (adjusted odds ratio: 4.30 (95% *CI*: 3.13, 5.90)). Otherwise, having over 20% of connected prescribers providing PSS in the prior month was associated with a large increase in the adjusted odds of PSS uptake (*aOR*:3.79 (3.15, 4.56), compared to no connections). Prescribers in Vancouver Coastal Health (1.31 (1.08, 1.59)) and Northern Health (1.30 (1.00, 1.70)) had higher adjusted odds of PSS uptake compared to Interior Health. OAT prescribers were 3 times more likely to provide PSS (3.35 (2.84, 3.95)). Less experienced prescribers were more likely to initiate PSS; having over 22.4 years of experience was associated with lower odds of PSS uptake (0.59 (0.49, 0.71)) compared to those with less than 4.5 years of experience. Each percentage point increase in clients accessing social assistance was associated with a 2% increase in the adjusted odds of PSS uptake. Treating a client with a COVID-19 diagnosis was associated with an increase in PSS uptake (1.50 (1.14, 1.99)). Each additional prescriber seen by a prescribers’ average client associated with a 38% increase in the odds of PSS dispensation (1.38 (1.25, 1.52)).

**Table 2:** Multiple logistic regression results for probability of PSS initiation: May 2020–August 2021

	Adjusted odds ratio	95% *CI*
Study month	0.97	(0.96, 0.99)
**Network characteristics**		
Percentage of connected physicians that prescribed PSS prior months		
*None*	Reference	
*10% or less*	1.77	(1.39, 2.25)
> *10 to 20%*	2.42	(1.97, 2.96)
> *20%*	3.79	(3.15, 4.56)
Percentage of connected physicians that are also connected	1.00	(0.99, 1.00)
Number of other prescribers their average clients received prescription dispensations from	1.38	(1.25, 1.52)
**Practice characteristics**		
Primary health authority practice location		
*Interior*	Reference	
*Fraser*	0.78	(0.63, 0.96)
*Vancouver Coastal*	1.31	(1.08, 1.59)
*Vancouver Island*	1.06	(0.86, 1.31)
*Northern*	1.30	(1.00, 1.70)
*Unknown*	0.61	(0.27, 1.37)
Prescriber speciality		
*General practice*	Reference	
*Nurse practitioner*	1.84	(1.42, 2.38)
*Psychiatry*	1.41	(1.10, 1.80)
*Known but other specialties*	1.46	(1.01, 2.12)
*Unknown (physician)*	0.47	(0.35, 0.62)
OAT prescriber	3.35	(2.84, 3.95)
**SUD prescribing history**		
Years since first billing record for a client with SUD/OUD		
< *4.5 years*	Reference	
*4.5 to* < *10.9 years*	0.74	(0.62, 0.89)
*10.9 to* < *22.4 years*	0.74	(0.62, 0.89)
≥ *22.4 years*	0.59	(0.49, 0.71)
**SUD client caseload**		
Number of SUD clients in the past 30 days		
*2 or less clients*	Reference	
*3 to 6 clients*	1.56	(1.15, 2.10)
*13 to 7 clients*	2.11	(1.53, 2.91)
*14 or more clients*	4.30	(3.13, 5.90)
Treated a client with a COVID-19 diagnosis	1.50	(1.14, 1.99)
Percentage of clients aged 40 or over	1.00	(0.99, 1.00)
Percent SUD/OUD clients with a CCI over 1	1.00	(0.99, 1.00)
Percentage of clients prescribed benzodiazepines	0.99	(0.99, 1.00)
Percentage of clients accessing social assistance during follow-up	1.02	(1.01, 1.02)
Percentage of clients experienced an overdose in the past 12 months	1.01	(1.00, 1.01)

*Abbreviations*: *PSS* prescribed safer supply, *OAT* opioid agonist treatment, *SUD* non-opioid substance use disorder, *OUD* opioid use disorder, *CCI* Charlson comorbidity index

Sensitivity analyses demonstrated the robustness of these findings (Tables A6–8). While estimates were different when stratified by PSS medication type, they were consistent with the main analysis (Table A9). Each medication type had the same gradient observed on the increased proportion of peer’s uptake of PSS which continually had stronger associations with an individual’s odds of PSS uptake. However, for benzodiazepine prescribing, while any peers had a significant impact on the odds, the magnitude was smaller than other PSS medications. When restricting the analysis based on SUD client load, the results were still robust (Tables A10–11). Requiring prescribers to have at least five SUD clients did increase the magnitude of the estimated effect of the network exposure. The impact of the PSS prescription classification may reduce the impact of the network exposure and SUD client load as when identifying PSS prescriptions using the more specific algorithm (Table A12), the magnitude of the network exposure and SUD client load were increased compared to the main analysis. Finally, when ending monthly measurements on the 15th of each month, we found the relationship with the study month was reversed (Table A13). There was also a slight increase in the estimated associations with the network exposure and SUD client load.

### Multiple regression analysis on PSS discontinuation

A total of 9678 prescriber months were included in the analysis **(**Table 3). Similar to PSS adoption, prescribers with larger SUD client loads and more connected peers dispensing PSS were strongly associated with discontinuation of PSS. When compared to treating 18 or less SUD clients, treating 86 or more clients was associated with an 85% reduction in the odds of discontinuing PSS (adjusted *OR*: 0.15, 95% *CI*: (0.11, 0.20)). Prescribers with over 20% of their peers dispensing PSS were less likely to discontinue PSS compared to clinicians with no peers dispensing PSS (0.29 (0.22, 0.38)). Otherwise, prescribers practicing in Fraser Health were the only location more likely to discontinue providing PSS compared to those practicing in Interior Health (1.36 (1.03, 1.79)), controlling for other factors. Prescribers with more years of experience treating SUD clients were also more likely to end PSS dispensations (≥ 17.4 years: 1.54 (1.24, 1.90), 8.3 to < 17.4 years: 1.26 (1.02, 1.55), 4 to < 8.3 years: 1.08 (0.87, 1.34) compared to < 4 years of experience). Unlike PSS uptake, differences between prescribing specialties compared to general practice physicians for discontinuation of PSS had estimates that were compatible with both increased and decreased odds of discontinuation of PSS.

**Table 3:** Logistic regression results for discontinuation of PSS prescriptions: July 2020–August 2021

	Adjusted odds ratio	95% *CI*
Study month	1.00	(0.98, 1.01)
**Network characteristics**		
Percentage of connected clinicians that prescribed PSS prior month		
*None*	Reference	
*10% or less*	0.87	(0.69, 1.10)
> *10 to 20%*	0.52	(0.41, 0.66)
> *20%*	0.29	(0.22, 0.38)
Percentage of connected clinicians that are also connected	1.00	(1.00, 1.01)
Number of other prescribers their average clients received prescription dispensations from	0.95	(0.84, 1.07)
**Practice characteristics**		
Primary health authority practice location		
*Interior*	Reference	
*Fraser*	1.36	(1.03, 1.79)
*Vancouver Coastal*	0.95	(0.73, 1.24)
*Vancouver Island*	1.06	(0.82, 1.37)
*Northern*	1.12	(0.80, 1.58)
*Unknown*	0.48	(0.15, 1.53)
Prescriber speciality		
*General practice*	Reference	
*Nurse practitioner (nonphysician)*	0.86	(0.65, 1.13)
*Psychiatry*	1.29	(0.95, 1.74)
*Known but other specialties*	1.22	(0.87, 1.71)
*Unknown (physician)*	1.21	(0.89, 1.64)
OAT prescriber	0.91	(0.75, 1.11)
**SUD prescribing history**		
Years since first billing record for a client with SUD/OUD		
< *4 years*	Reference	
*4 to* < *8.3 years*	1.08	(0.87, 1.34)
*8.3 to* < *17.4 years*	1.26	(1.02, 1.55)
≥ *17.4 years*	1.54	(1.24, 1.90)
**SUD caseload**		
Number of SUD clients in the past 30 days		
*18 or less clients*	Reference	
*18 to 43 clients*	0.42	(0.35, 0.51)
*44 to 85 clients*	0.20	(0.15, 0.26)
*86 or more clients*	0.15	(0.11, 0.20)
Treated a client with a COVID-19 diagnosis	0.87	(0.69, 1.11)
Percentage of clients aged 40 or over	1.00	(1.00, 1.01)
Percent SUD/OUD clients with a CCI over 1	1.00	(0.99, 1.00)
Percentage of clients prescribed benzos	1.00	(0.99, 1.00)
Percentage of clients accessing social assistance during follow-up	1.00	(0.99, 1.00)
Percentage of clients experienced an overdose in the past 12 months	1.00	(0.99, 1.01)

*Abbreviations*: *PSS* prescribed safer supply, *OAT* opioid agonist treatment, *SUD* non-opioid substance use disorder, *OUD* opioid use disorder, *CCI* Charlson comorbidity index

## Discussion

We evaluated the diffusion and adoption of a prescribed safer supply program implemented in BC and found that prescribers with larger SUD client load and higher proportions of peers that previously provided dispensations under PSS associated with increased odds of uptake of the PSS program. The importance of peer effects in the PSS adoption decision was reinforced by our findings on PSS discontinuation, as those with more peers who were still prescribing PSS were much less likely to discontinue dispensations. While risk or case of COVID-19 was eligibility criteria for PSS prescription, indications of diagnosis within clinicians’ case mix were only modestly positively associated with PSS adoption, thus further reinforcing network influences as the dominant. Overall adoption of the program was limited to 9.1% of all indicated prescribers, with 57% of prescribers who initiated PSS discontinuing dispensations later in the study period, and 63% of all early adopters forgoing PSS. Passive dissemination strategies may have been insufficient for subsequent adopters, those with lower client loads, and less peer prescribers knowledge updates and client’s needs weren’t met by the guideline or by the timeliness of available resources [48, 49].

Of critical importance in this application is the fact that the risk mitigation guidance was not explicitly recommending evidence-based practices [2]. This guidance was implemented on an emergency basis to protect against an anticipated, and ultimately observed, escalation in toxic drug-related deaths due to potential disruptions in illicit drug supply channels, guidance for self-isolation which could lead to a higher incidence of using alone, closures of supervised consumption facilities, and restricted access to other forms of treatment and care [50]. These findings nevertheless align with studies from other disease areas which demonstrate that the prescribing behaviors of a physician are impacted by the prescribing behaviors of their peers [27, 28, 51]. Aside from barriers in the outer context, adoption can still be hindered due to the inner context [52], systematic reviews have identified prescriber knowledge, attitudes and beliefs can hinder the success of implementation strategies for clinical practice guidelines [53], and attitudes and beliefs were no doubt influential in this particular application. The regulatory institution for physicians in the province expressed concerns regarding the limited empirical evidence base; the possibility of diversion, limited training, and expertise in providing prescribed safer supply within their scope of practice; and preference for team-based practices, which may deter adoption of new guidelines among prescribers in preference of well-established treatments [54–56]. As the evidence base for PSS emerges, future implementation strategies to improve PSS uptake will require engagement with both regulatory institutions and prescribers, acknowledging and addressing the concerns of both parties [57].

Our findings nevertheless highlight critical considerations for future implementation strategies on new policies for the clinical management of substance use disorders, which would benefit from leveraging or targeting networks of prescribers. First, our findings demonstrated prescribers who had a greater number of SUD clients were more likely to initiate PSS, suggesting these clinicians may have had greater awareness of announcements related to innovations in SUD care. Prescribers with smaller SUD client loads therefore may be harder to reach with passive dissemination strategies such as online educational materials and didactic lectures to learn about new clinical guidance and may require more active dissemination strategies such as academic detailing [24, 25] or interactive educational meetings [8].

Our findings provide further evidence that diffusion of clinical practice requires social reinforcement and draws attention to the need to identify which prescribers to target in dissemination strategies [27]. Recognizing this impact of diffusion among peers, and those on the outer edges of the network who are unlikely to be impacted by peers, emphasizes the need to consider differential strategies to engaged clinicians in larger and less-connected practice settings, respectively. Though some evidence is available on the effectiveness of specific singular active strategies [53, 58, 59], multifaceted implementation strategies, which include a combination of approaches such as educational changes, reminder systems, and organizational shifts for multidisciplinary collaboration, have demonstrated effectiveness [53, 59] in successfully disseminating guideline and policy changes in other disease areas. Future policy changes and clinical guideline updates should employ several different strategies to engage prescribers. Such strategies are particularly urgent in the present context given the pervasiveness of the toxic drug supply public health emergency, first issued in 2016 [60], which has increased in intensity due in large part to the changing composition and increasing potency of the illicit drug supply [61]. These changes in the underlying disease will require ongoing updates and revisions to clinical practice, which may otherwise suffer from waning attention with more frequent communications.

This study was not without limitations. Though we used population-based administrative data with true population coverage within the province of British Columbia, regardless of prescription drug insurance status, there was potential for some misclassification, particularly in identifying location as the location of prescriber and location of their clients may not coincide and clients may live in other health authorities. As with any observational study, our inferences may be subject to unmeasured confounding. In particular, we were unable to observe clinicians’ motivations for uptake and discontinuation. However, we hypothesize that motivations would be mediators on the causal path between peer influence and prescribing medications as individuals’ motivations will be influenced by their peers’ decisions, motivations, and opinions and therefore believe our interpretations were unaffected by these omissions. We otherwise do not observe a range of individual-specific characteristics of clinicians, including their year of graduation. Our constructed measures of the number of years of experience treating SUD clients likely adjusted for much of these potential confounding effects. Furthermore, though we had the number of positive COVID-19 cases within a prescriber’s caseload, we did not have information on who would have been isolating due to a close contact being positive, thus potentially diminishing the influence of COVID-19 on PSS uptake. Finally, while we have captured PSS implementation at the population level in the province of British Columbia, Canada, BC remains the only setting in Canada or elsewhere to have implemented such a program; if other settings choose to implement a prescriber-based safer supply program, both structural and epidemiological conditions may influence PSS uptake. The transportability of these results to other settings should be considered carefully.

## Conclusion

Our study demonstrates limited uptake and strong network influences in the decision to adopt a novel intervention among prescribers treating people who use substances in British Columbia, Canada. The passive dissemination employed had some success in the implementation of PSS; however, more is needed to engage individuals further out in the network with less SUD experience and peers.

### Supplementary Information

**Additional file 1: Supplementary Appendix. Table A1.** Databases used to construct the cohort. **Table A2.** Drug identification numbers for identification of opioid agonist treatment from PharmaNet. **Table A3.** Diagnostic codes used for opioid and non-opioid substance use disorders to identify substance use disorder clients. **Table A4.** Drug identification numbers for identification of possible prescribed safer supply. **Table A5.** Algorithms to identify prescribed safer supply prescriptions. **Table A6.** Logistic regression results for probability of PSS uptake between May 1st 2020 – August 31st 2021, with the PSS peer exposure redefined as proportion of patients shared with PSS prescribing peers’. **Table A7.** Logistic regression results for probability of PSS uptake with a longer lagged period: July 1st 2020 – August 31st 2021. **Table A8.** Logistic regression results for probability of PSS uptake between May 1st 2020 – August 31st 2021, with an additional exposure controlling for PSS prescribers with 2 degrees of separation. **Table A9.** Logistic regression results for probability of uptake of different PSS medication times between May 1st 2020 – August 31st 2021. **Table A10.** Logistic regression results for probability of PSS uptake for prescribers with more than one client with substance use disorder in the month prior. **Table A11.** Logistic regression results for probability of PSS uptake for prescribers with at least five clients with a substance use disorder in the month prior. **Table A12.** Logistic regression results for probability of PSS uptake under the more specific case-finding algorithm. **Table A13.** Logistic regression results for probability of PSS uptake when ending the calendar month on the 15th of each month.

## Data Availability

The datasets generated and/or analyzed during the current study are not publicly available due to privacy restrictions and concerns on individual-level administrative data. Materials and code available on request.

## Abbreviations

aOR: Adjusted odds ratio
BC: British Columbia
CCI: Charlson comorbidity index
CDS: Clarks chronic disease score
CI: Confidence interval
HA: Health authority
IQR: Interquartile range
OAT: Opioid agonist treatment
PSS: Prescribed safer supply
SNA: Social network analysis
SUD: Substance use disorder
