# Supplementary material for: Influence of physician networks on the implementation of pharmaceutical alternatives to a toxic drug supply in British Columbia
Source: Implement Sci. 2024 Jan 6;19:3. doi: 10.1186/s13012-023-01331-x (PMC10771688; doi:10.1186/s13012-023-01331-x)
Supplement: Supplementary file 1 — Additional file 1: Supplementary Appendix. Table A1. Databases used to construct the cohort. Table A2. Drug identification numbers for identification of opioid agonist treatment from PharmaNet. Table A3. Diagnostic codes used for opioid and non-opioid substance use disorders to identify substance use disorder clients. Table A4. Drug identification numbers for identification of possible prescribed safer supply. Table A5. Algorithms to identify prescribed safer supply prescriptions. Table A6. Logistic regression results for probability of PSS uptake between May 1st 2020 – August 31st 2021, with the PSS peer exposure redefined as proportion of patients shared with PSS prescribing peers’. Table A7. Logistic regression results for probability of PSS uptake with a longer lagged period: July 1st 2020 – August 31st 2021. Table A8. Logistic regression results for probability of PSS uptake between May 1st 2020 – August 31st 2021, with an additional exposure controlling for PSS prescribers with 2 degrees of separation. Table A9. Logistic regression results for probability of uptake of different PSS medication times between May 1st 2020 – August 31st 2021. Table A10. Logistic regression results for probability of PSS uptake for prescribers with more than one client with substance use disorder in the month prior. Table A11. Logistic regression results for probability of PSS uptake for prescribers with at least five clients with a substance use disorder in the month prior. Table A12. Logistic regression results for probability of PSS uptake under the more specific case-finding algorithm. Table A13. Logistic regression results for probability of PSS uptake when ending the calendar month on the 15th of each month. [file 13012_2023_1331_MOESM1_ESM.docx]

**Supplementary Appendix. *Influence of physician networks on the implementation of pharmaceutical alternatives to a toxic drug supply in British Columbia.***

*A1.1 Data linkage*

In British Columbia (BC), health insurance is publicly funded and applies to almost all residents of BC. Each resident has a unique personal healthcare number (PHN) tied to the individual and which is used when accessing the healthcare system. With the PHN, each resident’s access to the healthcare system is tracked between different points of healthcare contacts, specifically hospitalizations (Discharge abstract database), physicians visits (Medical service plan database), child births (Perinatal Data Registry), new prescriptions or refills at a pharmacy (PharmaNet), death records (Vital Stats and BC coroners data, ) and COVID-19 lab tests (BCCDC Covid-19 database and PLIS database). Detailed database descriptions are available in **Table A1**. Each database contains de-identified study specific IDs that are tied to an individual’s PHN, and this study ID is used to link personal records between the different databases, including BC provincial correction records. This link between BC provincial corrections is possible as the PHN is used for each individual for required health assessments within 24 hours of admission(1) and is therefore tied to the incarceration records, as the health care received while incarcerated is still part of the provincial system. Further healthcare received while incarcerated is also tracked through the PHN.

*A1.2. Prescribed safer supply prescription identifications.*

Dispensations of prescribed safer supply (PSS) were identified from PharmaNet database. As new DINs were not assigned for PSS medications, we developed algorithms to identify PSS recipients by applying restrictions to our case searches using prescription data including drug type prescription history, timing, and a list of keywords from the free-form codes up to 80 characters written in the ‘directions for use’ variable. This ‘directions for use’ variable is a field for instructions on how to use medications as prescribed from the physician. From possible PSS dispensation records based on drug type (**Table A4**), we constructed PSS episodes using the ‘service date’ and ‘days supplied’ fields in the PharmaNet database. Continuous PSS episodes had no interruptions in prescribed doses lasting ≥ 7 days(2) . An episode with at least one indication of PSS keywords (**Table A5**) was considered as PSS prescription.

Clinicians attached to a prescription via the de-identified prescriber ID field in PharmaNet within a PSS episode were defined as a PSS prescriber.

*A1.3 Positive COVID lab testing.*

There were several duplicate lab data for individuals. We only considered the first test as a positive diagnosis and excluded positives where the consecutive test was done within 90 days from the positive case. After 90 days it was considered a new positive. This was based on Canadian guidance suggesting that positive tests up to 3 months after may not represent a true re-infection(3).

**Table A1.** Databases used to construct the cohort.

| **Database** | **Description** | **Generating process** | **Key content** |
| --- | --- | --- | --- |
| PharmaNet | All prescriptions for drugs and medical supplies dispensed from pharmacies including hospital outpatient dispensations. | Electronically submitted by pharmacists dispensing medications in real time. Required for reimbursement. | Drugs dispensed (using DIN/PIN number), date of dispensation, quantity and duration of prescription, billing information, **prescriber code** and drug costs. |
| Discharge Abstract Database (DAD) | All hospital discharges, day surgery, transfers, and deaths of inpatients. Data of BC residents treated at hospital out of province, and out-of-province residents treated within BC hospitals included. | Data files grouped into fiscal years by separation date (not admission date). Each hospital submits electronic records of client visits to the provincial government which cleans and then submits the records to the Canadian Institute for Health Information (CIHI). CIHI regularly conducts re-abstraction to ensure data quality. | Hospitalization dates, most responsible diagnosis (ICD 9/10 code) and up to 24 additional diagnostic codes, 25 procedure codes using CCI/CCP procedure/ intervention codes^†^, transport method, transfers, **primary physician responsible** for stay, condition specific resource intensity weights, inpatient grouping. Hospital number, level of care, admission date/time, admission category, readmission, and transfer codes, discharge date/time, discharge, disposition, length of stay, stay by level of care. |
| Medical Services Plan (MSP) Database | All medically necessary services provided by fee-for-service practitioners covered by the province’s universal insurance program: Medical Services Plan (MSP). | Majority of billing records submitted electronically by practitioners’ offices for reimbursement purposes. Diagnosis codes accurate only to 3^rd^ digit. | Medically necessary services including laboratory and diagnostic procedures (x-rays, ultrasounds), and dental and oral surgery performed in hospital. Up to 5 diagnoses codes included (ICD-9). Service date, fee item, diagnostic codes, **practitioner code**, service costs and location. |
| Vital Statistics (VS) | All deaths registered in the province. | Data is checked against nationally uniform vital registration and statistics standards. | Date of death (year and month), location, underlying cause of death (ICD-9 and ICD-10), and nature of injury codes. |
| BC Coroners Service  (BCCS) | All unnatural, sudden and unexpected, unexplained or unattended deaths in British Columbia | The agency maintains a database and conducts ongoing surveillance of common causes and circumstances of death. | Date of death, location of injury, cause of unnatural death, mode of consumption, post mortem toxicology |
| National Ambulatory Care Reporting System (NACRS) | All emergency department visits | The centralized data processing of the NACRS records, done by the Canadian Institute of Health Information (CIHI), results in increased efficiency and  standardization among the participating provinces. | Institution/hospital number; triage, registration, assessment and disposition date/time; presenting complaint codes; ED discharge diagnosis code. |
| Perinatal Data Registry (PDR) | Maternal and child health for all provincial births from | Perinatal data is collected from facilities throughout the province and imported into the central BC Perinatal Data  Registry (BCPDR). | Demographics of mother, past obstetric history, information on current pregnancy, labour and delivery, post-delivery, diagnoses and procedures; demographics of baby, information on birth and care, diagnoses and procedures |
| BC Social Development and Poverty Reduction  (SDPR) | Income Assistance benefits for qualifying people in British Columbia | Data is extracted from the Ministry of Social Development and Poverty Reduction’s records of social assistance or disability assistance payments that have been made to individuals under the BC Employment and Assistance program. | Program name, age, family and dependent information, total payment received, indicators for no fixed address, methadone use, crisis grants, and other income received. |
| BC Provincial Corrections | Individuals incarceration in provincial prisons (sentences of ≤ 2 years) | All adults corrections data received from the Ministry of Public Safety and Solicitor General (PSSG) | Inmate client information, dates and times of admissions and release |
| BCCDC Covid-19 positive cases database | Individuals with a positive COVID-19 case | All individuals who were diagnosed via lab test. | Individual information, positive case report date. |
| Provincial Laboratory Information Solution COVID-19  database | Lab specimens for COVID-19 test | All individuals who obtained a lab-based COVID-19 test. | Individual information, test result (positive, negative or indeterminate), test date. |

*Abbreviations:* DIN: Drug Identification Number; PIN: Product Identification Number; ^†^ Coding structures used by the Canadian Institute of Health Information (CIHI); ^‡^ A standardized code picklist for presenting complaint developed by CIHI.

**Table A2.** Drug identification numbers for identification of opioid agonist treatment from PharmaNet

| **OAT** | **DIN/PIN*** |
| --- | --- |
| Methadone | 999792, 999793, 66999990, 66999991, 66999992, 66999993, 66999997, 66999998, 66999999, 67000000, 67000001, 67000002, 67000003, 67000004, 67000005, 67000006, 67000007, 67000008, 67000009,67000010, 67000011, 67000012, 67000013, 67000014, 67000015, 67000016, 67000017, 67000018, 67000019, 67000020 |
| Buprenorphine/naloxone | 2295695, 2295709, 2408090, 2408104, 2424851, 2424878, 2453908, 2453916, 2468085, 2468093 |
| Slow-release oral morphine (Kadian) | 22123349, 22123346, 22123347, 22123348 |
| Injectable OAT^†^ | 2146126, 22123340, 22123357, 66123367 |
| Tablet OAT (Hydromorphone) | 786543, 885428 |

*Drug Identification Numbers (DIN)/Product Identification Numbers (PIN); ^†^Diacetylmorphine or hydromorphone with some restrictions based on prescriber, dispensing pharmacy and/or date.

**Table A3.** Diagnostic codes used for opioid and non-opioid substance use disorders to identify substance use disorder clients.

| **Diseases** | **Diagnostic code** | **References** |
| --- | --- | --- |
| Opioid use disorder* | ICD-9 from DAD and MSP: 304.0, 304.7, 305.5, 965.0, E850.0  ICD-10 from DAD and NACRS: F11, T40.0 – T40.4, T40.6  Indication of opioid use disorder during the pregnancy from BCPDR. Receipt of any OAT medication DIN/PIN in Table A8. | (4) |
| Substance use disorder*^‡^ | ICD-9 from DAD and MSP: 292, 304.x (1-6,8,9), 305.x (2-4,6-9), 655.5, 967, 969.x (4,6,7), 970, E851, E852, E853.2, E854.x (1,2,3), V65.42; ICD-10 from DAD and NACRS: F12-F16, F19, X42, X62, Y12, T40.5, T40.7-T40.9, T42.4, T43.6, Z50.3, Z71.5, Z72.2; indication of substance use during the pregnancy from BCPDR | (5), (6) |

*Abbreviations:* ICD: International Classification of Diseases; DAD: Discharge Abstract Database (records of hospitalizations); NACRS: National Ambulatory Care Reporting System (records of emergency visits); MSP: Medical Service Plan (physician billing records); BCPDR: BC Perinatal data registry. *To minimize misclassification due to errors in the coding of physician billing records, we applied a case finding algorithm based on the presence of at least 1 hospitalization, ED visit, BCPDR record, more than 3 physician billing records, or medication receipt for alcohol use disorder; ^†^Any indication of depression, anxiety, psychotic illness, personality disorders, attention-deficit/hyperactivity disorders, or bipolar disorders; ^‡^Any indication of non-opioid drug use, poisoning (accidental or intentional), or substance use counselling or rehab, excluding alcohol use disorder;

**Table A4.** Drug identification numbers for identification of possible prescribed safer supply

| **Drug type/drug sub-type** | **Drug Identification Numbers DIN/PINs** |
| --- | --- |
| ***Opioids*** | |
| Hydromorphone | 2364158, 786543, 2225255, 2192144, 2245705, 2337274, 885428, 2319446 |
| M-Eslon | 2019930, 2019949, 2019957, 2019965, 2177749, 2177757 |
| ***Stimulants*** | |
| Dextroamphetamine | 2448319, 2448327, 2481464, 2481472, 181439, 181447, 1924559, 1924567, 27065, 1924516, 2443236 |
| Methylphenidate | 2441934, 2441942 ,2441950, 2441969,2249324, 2249332, 2273950, 2330377, 2452731, 2452758, 2452766, 2266687,2326221, 2326248, 2326256, 2243222, 422975, 422983, 2126486, 2126494, 2246991, 584991, 585009, 2234749, 2413728, 2413736, 2413744, 2413752, 2230321, 2230322, 2247364, 5606, 5614, 5185, 632775, 2320312, 2315068, 2315076, 2315084, 2315092 |
| ***Benzodiazepines*** | |
| Diazepam | 2247173, 2247174, 2247176, 2238162, 362158, 405329, 405337, 434388, 434396, 313580, 2243240, 2386143, 399728, 2385392, 466891, 466905, 303461, 396230, 2137399, 272639, 272647, 280429, 276650, 276642, 272450, 272434, 272442,2247490, 2247491, 2247492, 891797, 13293, 13277, 13285, 12874, 13110, 13757, 13765, 13773, 2065614, 2005492,  602825, 22123106 |
| Clonazepam | 2365243, 2365251, 2365278, 2177889, 2177897, 2230366, 2230368, 2230369, 2344602, 2344610, 2442027, 2442035, 2442043, 2442051, 2340968, 2235370, 2237277, 2340976, 2235379, 2237278, 2220598, 2220601, 2344629, 2270641, 2270668, 2270676, 2130998, 2131013, 2131005, 2224100, 2230950, 2230951, 2173344, 2173352, 2236947, 2145227, 2145235, 2145243, 2236948, 2179660, 2048701, 2048728, 2048736, 2207818, 2311593, 2311607, 2311615, 2103656, 2103737, 2242077, 2242078, 382825, 382841, 2233960, 2233982, 2233985, 2239024, 2239025, 2303310, 2303329, 2303337, 2345676, 2303302 |

**Table A5.** Algorithms to identify prescribed safer supply prescriptions.

| **Opioids**  **Case Definition 1: Higher specificity lower sensitivity**  For each chemical type, person NOT on PSS medication in the 2 months prior to March 27, 2020, but prescribed medication from March 27, 2020 with one of the keywords (i.e. substring) occurred in the directions for use variable: *corona, cov (*excluding *cover), crisis, mitigat, pand (*for *pandemic), pprm, pwm, risk m, riskmitigation, safe s (*for *safe suppl), safer s, safe drug su, safe+supply, safe consum, clean drug supply, fentan.*  **Case Definition 2: Higher sensitivity, lower specificity**  For each chemical type, person NOT on RMG medication in the 2 months prior to March 27, 2020, but prescribed medication from March 27, 2020 with one of the keywords (i.e. substring) occurred in the directions for use variable as listed in definition 1 or any of the following substring in the directions for use variable: *carr, crav, opioid c* (for *opioid craving*)*, del* (for *delivery*)*, distancing, guidan, guideline, illici, interim, isolatio, management, outbreak, overdo, opioid replacement, risk, saf, OAT/s, OAT sa, unwit, no w* (for *no witness*)*, not wi, withd, withrawal, relieve wi, needed for w, pwd, opioid w, opiate w, with wi, witness, wittness, dw, first dose w, first dose to be wit, one dose w, 1st w, harm* (excluding *pharm*).  *Exclusion: “pain” in directions for use variable |
| --- |
| **Stimulants**  **Case Definition 1: Higher specificity lower sensitivity**  For each chemical type, person NOT on PSS medication in the 2 months prior to March 27, 2020, but prescribed medication from March 27, 2020 with one of the keywords (i.e. substring) occurred in the directions for use variable: *corona, cov (*excluding *cover), crisis, mitigat, pand (*for *pandemic), pprm, pwm, risk m, riskmitigation, safe s (*for *safe suppl), safer s, safe drug su, safe+supply, safe consum, clean drug supply.*  **Case Definition 2: Higher sensitivity, lower specificity**  For each chemical type, person NOT on RMG medication in the 2 months prior to March 27, 2020, but prescribed medication from March 27, 2020 with one of the keywords (i.e. substring) occurred in the directions for use variable as listed in definition 1 or any of the following substring in the directions for use variable: *carr, crav, del* (for *delivery*)*, distancing, guidan, guideline, illici, interim, isolatio, management, outbreak, overdo, replace + meth, risk, saf, unwit, no w* (for *no witness*)*, not wi, withd, withrawal, relieve wi, needed for w, pwd, pain/wit, with wi, witness, wittness, dw, first dose w, first dose to be wit, one dose w, 1st w, harm* (excluding *pharm*). |
| **Benzodiazepines**  **Case Definition 1: Higher specificity lower sensitivity**  For each chemical type, person NOT on PSS medication in the 2 months prior to March 27, 2020, but prescribed medication from March 27, 2020 with one of the keywords (i.e. substring) occurred in the directions for use variable: *corona, cov (excluding* cover*), crisis, mitigat, pand (for pandemic), pprm, pwm, risk m, riskmitigation, safe s (for safe suppl), safer s, safe drug su, safe+supply, safe consum, clean drug supply.*  **Case Definition 2: Higher sensitivity, lower specificity**  For each chemical type, person NOT on RMG medication in the 2 months prior to March 27, 2020, but prescribed medication from March 27, 2020 with one of the keywords (i.e. substring) occurred in the directions for use variable as listed in definition 1 or any of the following substring in the directions for use variable: *carr, crav, benz, del* (for *delivery*)*, distancing, guidan, guideline, illici, interim, isolatio, management, outbreak, overdo, risk, saf, unwit, no w* (for *no witness*)*, not wi, withd, withrawal, relieve wi, needed for w, pwd, pain/wit, with wi, witness, wittness, dw, first dose w, first dose to be wit, one dose w, 1st w, harm* (excluding *pharm*).  *Exclusion: “alcohol” or “CIWA” in directions for use variable. |

**Table A6.** Logistic regression results for probability of PSS uptake between May 1^st^ 2020 – August 31^st^ 2021, with the PSS peer exposure redefined as proportion of patients shared with PSS prescribing peers’.

|  | Odds ratio | 95% CI |
| --- | --- | --- |
| Study month | 0.97 | (0.96, 0.98) |
| **Network characteristics** |  |  |
| Percentage of all shared clients to peers’ that prescribed PSS prior months |  |  |
| *None* | Reference |  |
| *10% or less* | 1.75 | (1.38, 2.22) |
| *>10% to 20%* | 2.31 | (1.87, 2.85) |
| *>20%* | 3.85 | (3.21, 4.63) |
| Percentage of connected physicians that are also connected | 1.00 | (0.99, 1.00) |
| Number of additional physicians their average clients saw | 1.38 | (1.25, 1.53) |
| **Practice characteristics** |  |  |
| Primary HA practice |  |  |
| *Interior* | Reference |  |
| *Fraser* | 0.80 | (0.65, 0.98) |
| *Vancouver Coastal* | 1.32 | (1.09, 1.61) |
| *Vancouver Island* | 1.08 | (0.88, 1.34) |
| *Northern* | 1.32 | (1.01, 1.72) |
| *Unknown* | 0.63 | (0.28, 1.41) |
| Physician speciality |  |  |
| *General practice* | Reference |  |
| *Nurse practitioner (non-physician)* | 1.83 | (1.41, 2.38) |
| *Psychiatry* | 1.38 | (1.08, 1.76) |
| *Known but other specialties* | 1.45 | (1.00, 2.11) |
| *Unknown (physician)* | 0.47 | (0.35, 0.63) |
| OAT prescriber | 3.32 | (2.82, 3.92) |
| **SUD prescribing history** |  |  |
| Years since first billing record for a client with SUD/OUD |  |  |
| *<4.5 years* | Reference |  |
| *4.5 to <10.9 years* | 0.74 | (0.62, 0.89) |
| *10.9 to <22.4 years* | 0.74 | (0.62, 0.88) |
| *≥ 22.4 years* | 0.59 | (0.49, 0.71) |
| **SUD client caseload** |  |  |
| Number of SUD clients in the past 30 days |  |  |
| *2 or less clients* | Reference |  |
| *3 to 6 clients* | 1.56 | (1.16, 2.11) |
| *13 to 7 clients* | 2.13 | (1.54, 2.93) |
| *14 or more clients* | 4.28 | (3.12, 5.88) |
| Treated a client with a covid-19 diagnosis | 1.51 | (1.15, 1.98) |
| Percentage of clients aged 40 or over | 1.00 | (0.99, 1.00) |
| Percent SUD/OUD clients with a CCI over 1 | 1.00 | (0.99, 1.00) |
| Percentage of clients prescribed benzodiazepines | 0.99 | (0.99, 1.00) |
| Percentage of clients accessing social assistance during follow-up | 1.02 | (1.01, 1.02) |
| Percentage of clients experienced an overdose in the past 12-months | 1.01 | (1.00, 1.01) |

**Table A7.** Logistic regression results for probability of PSS uptake with a longer lagged period: July 1^st^ 2020 – August 31^st^ 2021.

|  | Odds ratio | 95% CI |
| --- | --- | --- |
| Study month | 0.98 | (0.97, 1.00) |
| **Network characteristics** |  |  |
| Percentage of connected peers that prescribed PSS prior months |  |  |
| *None* | Reference |  |
| *10% or less* | 1.54 | (1.18, 2.01) |
| *>10% to 20%* | 2.17 | (1.74, 2.70) |
| *>20%* | 3.25 | (2.67, 3.96) |
| Percentage of connected physicians that are also connected | 1.00 | (0.99, 1.00) |
| Number of additional physicians their average clients saw | 1.35 | (1.21, 1.50) |
| **Practice characteristics** |  |  |
| Primary HA practice |  |  |
| *Interior* | Reference |  |
| *Fraser* | 0.78 | (0.62, 0.97) |
| *Vancouver Coastal* | 1.23 | (1.00, 1.52) |
| *Vancouver Island* | 0.97 | (0.77, 1.21) |
| *Northern* | 1.25 | (0.94, 1.67) |
| *Unknown* | 0.59 | (0.24, 1.41) |
| Physician speciality |  |  |
| *General practice* | Reference |  |
| *Nurse practitioner (non-physician)* | 1.69 | (1.26, 2.26) |
| *Psychiatry* | 1.49 | (1.15, 1.93) |
| *Known but other specialties* | 1.41 | (0.95, 2.10) |
| *Unknown (physician)* | 0.49 | (0.36, 0.67) |
| OAT prescriber | 3.20 | (2.67, 3.82) |
| **SUD prescribing history** |  |  |
| Years since first billing record for a client with SUD/OUD |  |  |
| *<4.5 years* | Reference |  |
| *4.5 to <10.9 years* | 0.73 | (0.60, 0.89) |
| *10.9 to <22.4 years* | 0.76 | (0.63, 0.93) |
| *≥ 22.4 years* | 0.56 | (0.45, 0.69) |
| **SUD client caseload** |  |  |
| Number of SUD clients in the past 30 days |  |  |
| *2 or less clients* | Reference |  |
| *3 to 6 clients* | 1.33 | (0.97, 1.82) |
| *13 to 7 clients* | 1.88 | (1.34, 2.63) |
| *14 or more clients* | 3.54 | (2.53, 4.94) |
| Treated a client with a covid-19 diagnosis | 1.55 | (1.18, 2.05) |
| Percentage of clients aged 40 or over | 1.00 | (0.99, 1.00) |
| Percent SUD/OUD clients with a CCI over 1 | 1.00 | (0.99, 1.00) |
| Percentage of clients prescribed benzodiazepines | 1.00 | (0.99, 1.00) |
| Percentage of clients accessing social assistance during follow-up | 1.01 | (1.01, 1.02) |
| Percentage of clients experienced an overdose in the past 12-months | 1.01 | (1.00, 1.01) |

**Table A8.** Logistic regression results for probability of PSS uptake between May 1^st^ 2020 – August 31^st^ 2021, with an additional exposure controlling for PSS prescribers with 2 degrees of separation.

|  | Odds ratio | 95% CI |
| --- | --- | --- |
| Study month | 0.97 | (0.95, 0.98) |
| **Network characteristics** |  |  |
| Percentage of connected physicians that prescribed PSS prior months |  |  |
| *None* | Reference |  |
| *10% or less* | 1.78 | (1.39, 2.27) |
| *>10% to 20%* | 2.41 | (1.94, 2.99) |
| *>20%* | 3.78 | (3.07, 4.65) |
| Percentage of physicians with 2 degrees of separation that prescribed PSS prior months |  |  |
| *None* | Reference |  |
| *10% or less* | 0.76 | (0.51, 1.14) |
| *>10% to 20%* | 0.88 | (0.59, 1.33) |
| *>20%* | 0.83 | (0.54, 1.28) |
| Percentage of connected physicians that are also connected | 1.00 | (0.99, 1.00) |
| Number of additional physicians their average clients saw | 1.39 | (1.25, 1.53) |
| **Practice characteristics** |  |  |
| Primary HA practice |  |  |
| *Interior* | Reference |  |
| *Fraser* | 0.75 | (0.60, 0.94) |
| *Vancouver Coastal* | 1.27 | (1.02, 1.57) |
| *Vancouver Island* | 1.03 | (0.82, 1.28) |
| *Northern* | 1.27 | (0.97, 1.66) |
| *Unknown* | 0.59 | (0.26, 1.33) |
| Physician speciality |  |  |
| *General practice* | Reference |  |
| *Nurse practitioner (non-physician)* | 1.83 | (1.41, 2.37) |
| *Psychiatry* | 1.41 | (1.10, 1.80) |
| *Known but other specialties* | 1.46 | (1.01, 2.12) |
| *Unknown (physician)* | 0.47 | (0.35, 0.63) |
| OAT prescriber | 3.34 | (2.83, 3.94) |
| **SUD prescribing history** |  |  |
| Years since first billing record for a client with SUD/OUD |  |  |
| *<4.5 years* | Reference |  |
| *4.5 to <10.9 years* | 0.74 | (0.62, 0.89) |
| *10.9 to <22.4 years* | 0.74 | (0.62, 0.89) |
| *≥ 22.4 years* | 0.59 | (0.48, 0.71) |
| **SUD caseload** |  |  |
| Number of SUD clients in the past 30 days |  |  |
| *2 or less clients* | Reference |  |
| *3 to 6 clients* | 1.61 | (1.19, 2.19) |
| *13 to 7 clients* | 2.21 | (1.58, 3.07) |
| *14 or more clients* | 4.50 | (3.24, 6.26) |
| Treated a client with a covid-19 diagnosis | 1.50 | (1.13, 1.98) |
| Percentage of clients aged 40 or over | 1.00 | (0.99, 1.00) |
| Percent SUD/OUD clients with a CCI over 1 | 1.00 | (0.99, 1.00) |
| Percentage of clients prescribed benzodiazepines | 0.99 | (0.99, 1.00) |
| Percentage of clients accessing social assistance during follow-up | 1.02 | (1.01, 1.02) |
| Percentage of clients experienced an overdose in the past 12-months | 1.01 | (1.00, 1.01) |

**Table A9.** Logistic regression results for probability of uptake of different PSS medication times between May 1^st^ 2020 – August 31^st^ 2021.

| Type of PSS | Opioid | Stimulant | Benzodiazepine |
| --- | --- | --- | --- |
|  | Odds ratio estimate (95% CI) | | |
| Study month | 0.95 (0.93, 0.97) | 0.90 (0.87, 0.92) | 0.96 (0.93, 0.98) |
| **Network characteristics** |  |  |  |
| Percentage of connected physicians that prescribed specific PSS prior months |  |  |  |
| *None* | Reference | Reference | Reference |
| *10% or less for opioids 5% or less for stimulants any for benzodiazepines* | 2.40 (1.82, 3.15) | 2.86 (1.84, 4.44) | 1.96 (1.53, 2.51) |
| *>10% to 15% for opioids >5% to 10% for stimulants* | 3.67 (2.78, 4.84) | 2.95 (2.11, 4.11) | ----- |
| *>15% for opioids > 10% for stimulants* | 4.77 (3.83, 5.93) | 4.06 (3.12, 5.28) | ----- |
| Percentage of connected physicians that are also connected | 1.00 (0.99, 1.00) | 1.00 (0.99, 1.00) | 0.99 (0.99, 1.00) |
| Number of additional physicians their average clients saw | 1.37 (1.22, 1.54) | 1.44 (1.25, 1.65) | 1.50 (1.27, 1.76) |
| **Practice characteristics** |  |  |  |
| Primary HA practice |  |  |  |
| *Interior* | Reference | Reference | Reference |
| *Fraser* | 0.76 (0.58, 0.99) | 0.65 (0.44, 0.95) | 1.03 (0.71, 1.49) |
| *Vancouver Coastal* | 1.50 (1.18, 1.91) | 1.93 (1.42, 2.63) | 1.52 (1.07, 2.16) |
| *Vancouver Island* | 1.13 (0.87, 1.47) | 1.16 (0.82, 1.64) | 1.38 (0.95, 2.00) |
| *Northern* | 1.51 (1.11, 2.06) | 1.32 (0.86, 2.04) | 0.64 (0.34, 1.21) |
| *Unknown* | 0.76 (0.30, 1.89) | 0.48 (0.11, 2.07) | 0.63 (0.14, 2.84) |
| Physician speciality |  |  |  |
| *General practice* | Reference | Reference | Reference |
| *Nurse practitioner (non-physician)* | 2.15 (1.59, 2.89) | 2.32 (1.63, 3.30) | 1.12 (0.68, 1.87) |
| *Psychiatry* | 0.96 (0.69, 1.33) | 1.27 (0.87, 1.86) | 1.39 (0.95, 2.03) |
| *Unknown (physician)* | 1.82 (1.17, 2.83) | 1.85 (1.06, 3.23) | 1.47 (0.79, 2.74) |
| *Known but other specialties* | 0.48 (0.34, 0.69) | 0.41 (0.25, 0.66) | 0.49 (0.30, 0.80) |
| OAT prescriber | 5.09 (4.05, 6.39) | 3.93 (2.97, 5.20) | 2.95 (2.25, 3.87) |
| **SUD prescribing history** |  |  |  |
| Years since first billing record for a client with SUD/OUD |  |  |  |
| *<4.5 years* | Reference | Reference | Reference |
| *4.5 to <10.9 years* | 0.75 (0.61, 0.93) | 0.56 (0.42, 0.75) | 0.70 (0.51, 0.95) |
| *10.9 to <22.4 years* | 0.71 (0.57, 0.88) | 0.56 (0.42, 0.74) | 0.70 (0.52, 0.94) |
| *≥ 22.4 years* | 0.55 (0.44, 0.70) | 0.53 (0.40, 0.72) | 0.53 (0.39, 0.74) |
| **SUD client caseload** |  |  |  |
| Number of SUD clients in the past 30 days |  |  |  |
| *2 or less clients* | Reference | Reference | Reference |
| *3 to 6 clients* | 1.30 (0.88, 1.93) | 1.75 (1.03, 2.99) | 2.48 (1.46, 4.21) |
| *13 to 7 clients* | 2.02 (1.33, 3.06) | 2.90 (1.63, 5.18) | 3.43 (1.95, 6.02) |
| *14 or more clients* | 4.14 (2.75, 6.24) | 6.52 (3.70, 11.47) | 6.59 (3.74, 11.61) |
| Treated a client with a covid-19 diagnosis | 1.73 (1.26, 2.38) | 1.13 (0.66, 1.93) | 1.32 (0.78, 2.23) |
| Percentage of clients accessing social assistance during follow-up | 1.02 (1.01, 1.02) | 1.02 (1.02, 1.03) | 1.02 (1.02, 1.03) |
| Percentage of clients prescribed benzodiazepines | 0.99 (0.99, 1.00) | 0.99 (0.98, 1.00) | 1.00 (0.99, 1.01) |
| Percent SUD/OUD clients with a CCI over 1 | 1.00 (0.99, 1.00) | 1.00 (0.99, 1.00) | 0.99 (0.98, 1.00) |
| Percentage of clients aged 40 or over | 1.00 (0.99, 1.00) | 1.00 (0.99, 1.00) | 0.99 (0.98, 1.00) |
| Percentage of clients experienced an overdose in the past 12-months | 1.00 (0.99, 1.01) | 1.00 (0.99, 1.01) | 1.02 (1.01, 1.02) |

**Table A10.** Logistic regression results for probability of PSS uptake for prescribers with more than one client with substance use disorder in the month prior.

|  | Odds ratio | 95% CI |
| --- | --- | --- |
| Study month | 0.97 | (0.96, 0.98) |
| **Network characteristics** |  |  |
| Percentage of connected physicians that prescribed PSS prior months |  |  |
| *None* | Reference |  |
| *10% or less* | 1.82 | (1.42, 2.33) |
| *>10% to 20%* | 2.48 | (2.00, 3.07) |
| *>20%* | 3.82 | (3.12, 4.67) |
| Percentage of connected physicians that are also connected | 1.00 | (0.99, 1.00) |
| Number of additional physicians their average clients saw | 1.44 | (1.29, 1.62) |
| **Practice characteristics** |  |  |
| Primary HA practice |  |  |
| *Interior* | Reference |  |
| *Fraser* | 0.76 | (0.61, 0.94) |
| *Vancouver Coastal* | 1.29 | (1.05, 1.58) |
| *Vancouver Island* | 1.02 | (0.82, 1.27) |
| *Northern* | 1.26 | (0.96, 1.64) |
| *Unknown* | 0.61 | (0.24, 1.51) |
| Physician speciality |  |  |
| *General practice* | Reference |  |
| *Nurse practitioner (non-physician)* | 1.72 | (1.32, 2.25) |
| *Psychiatry* | 1.26 | (0.98, 1.62) |
| *Known but other specialties* | 1.6 | (1.07, 2.38) |
| *Unknown (physician)* | 0.47 | (0.35, 0.63) |
| OAT prescriber | 3.09 | (2.61, 3.65) |
| **SUD prescribing history** |  |  |
| Years since first billing record for a client with SUD/OUD |  |  |
| *<4.5 years* | Reference |  |
| *4.5 to <10.9 years* | 0.77 | (0.64, 0.92) |
| *10.9 to <22.4 years* | 0.75 | (0.62, 0.9) |
| *≥ 22.4 years* | 0.61 | (0.5, 0.74) |
| **SUD caseload** |  |  |
| Number of SUD clients in the past 30 days |  |  |
| *2 clients* | Reference |  |
| *3 to 6 clients* | 1.42 | (0.99, 2.03) |
| *13 to 7 clients* | 2.05 | (1.4, 3.01) |
| *14 or more clients* | 4.29 | (2.93, 6.3) |
| Treated a client with a covid-19 diagnosis | 1.47 | (1.11, 1.96) |
| Percentage of clients aged 40 or over | 0.99 | (0.99, 1.00) |
| Percent SUD/OUD clients with a CCI over 1 | 1.00 | (0.99, 1.00) |
| Percentage of clients prescribed benzodiazepines | 1.00 | (0.99, 1.00) |
| Percentage of clients accessing social assistance during follow-up | 1.02 | (1.02, 1.02) |
| Percentage of clients experienced an overdose in the past 12-months | 1.01 | (1.00, 1.02) |

**Table A11.** Logistic regression results for probability of PSS uptake for prescribers with at least five clients with a substance use disorder in the month prior.

|  | Odds ratio | 95% CI |
| --- | --- | --- |
| Study month | 0.97 | (0.95, 0.98) |
| **Network characteristics** |  |  |
| Percentage of connected physicians that prescribed PSS prior months |  |  |
| *None* | Reference |  |
| *10% or less* | 2.01 | (1.53, 2.65) |
| *>10% to 20%* | 2.86 | (2.24, 3.67) |
| *>20%* | 4.17 | (3.27, 5.33) |
| Percentage of connected physicians that are also connected | 1.00 | (0.99, 1.00) |
| Number of additional physicians their average clients saw | 1.75 | (1.50, 2.05) |
| **Practice characteristics** |  |  |
| Primary HA practice |  |  |
| *Interior* | Reference |  |
| *Fraser* | 0.76 | (0.60, 0.96) |
| *Vancouver Coastal* | 1.26 | (1.01, 1.57) |
| *Vancouver Island* | 1.02 | (0.80, 1.29) |
| *Northern* | 1.30 | (0.99, 1.73) |
| *Unknown* | 0.50 | (0.17, 1.49) |
| Physician speciality |  |  |
| *General practice* | Reference |  |
| *Nurse practitioner (non-physician)* | 1.62 | (1.21, 2.17) |
| *Psychiatry* | 1.11 | (0.85, 1.46) |
| *Known but other specialties* | 1.44 | (0.82, 2.53) |
| *Unknown (physician)* | 0.35 | (0.23, 0.53) |
| OAT prescriber | 2.72 | (2.27, 3.25) |
| **SUD prescribing history** |  |  |
| Years since first billing record for a client with SUD/OUD |  |  |
| *<4.5 years* | Reference |  |
| *4.5 to <10.9 years* | 0.82 | (0.67, 1.00) |
| *10.9 to <22.4 years* | 0.80 | (0.66, 0.98) |
| *≥ 22.4 years* | 0.66 | (0.53, 0.82) |
| **SUD caseload** |  |  |
| Number of SUD clients in the past 30 days |  |  |
| *5 to 6 clients* | Reference |  |
| *13 to 7 clients* | 1.64 | (1.21, 2.24) |
| *14 or more clients* | 3.57 | (2.65, 4.82) |
| Treated a client with a covid-19 diagnosis | 1.49 | (1.11, 2.00) |
| Percentage of clients aged 40 or over | 0.99 | (0.99, 1.00) |
| Percent SUD/OUD clients with a CCI over 1 | 0.99 | (0.99, 1.00) |
| Percentage of clients prescribed benzodiazepines | 1.00 | (0.99, 1.00) |
| Percentage of clients accessing social assistance during follow-up | 1.02 | (1.02, 1.03) |
| Percentage of clients experienced an overdose in the past 12-months | 1.00 | (0.99, 1.02) |

**Table A12.** Logistic regression results for probability of PSS uptake under case-finding algorithm definition 1.

|  | Odds ratio | 95% CI |
| --- | --- | --- |
| Study month | 0.95 | (0.93, 0.97) |
| **Network characteristics** |  |  |
| Percentage of connected physicians that prescribed PSS prior months |  |  |
| *None* | Reference |  |
| *10% or less* | 2.40 | (1.81, 3.19) |
| *>10% to 20%* | 3.77 | (2.91, 4.88) |
| *>20%* | 5.78 | (4.50, 7.43) |
| Percentage of connected physicians that are also connected | 1.00 | (0.99, 1.00) |
| Number of additional physicians their average clients saw | 1.39 | (1.22, 1.57) |
| **Practice characteristics** |  |  |
| Primary HA practice |  |  |
| *Interior* | Reference |  |
| *Fraser* | 0.67 | (0.50, 0.90) |
| *Vancouver Coastal* | 1.56 | (1.21, 2.02) |
| *Vancouver Island* | 1.08 | (0.81, 1.43) |
| *Northern* | 1.47 | (1.05, 2.06) |
| *Unknown* | 0.64 | (0.21, 1.90) |
| Physician speciality |  |  |
| *General practice* | Reference |  |
| *Nurse practitioner (non-physician)* | 1.64 | (1.21, 2.22) |
| *Psychiatry* | 0.62 | (0.43, 0.90) |
| *Known but other specialties* | 1.79 | (1.12, 2.86) |
| *Unknown (physician)* | 0.41 | (0.28, 0.61) |
| OAT prescriber | 5.41 | (4.18, 7.00) |
| **SUD prescribing history** |  |  |
| Years since first billing record for a client with SUD/OUD |  |  |
| *<4.5 years* | Reference |  |
| *4.5 to <10.9 years* | 0.74 | (0.60, 0.92) |
| *10.9 to <22.4 years* | 0.62 | (0.49, 0.79) |
| *≥ 22.4 years* | 0.53 | (0.41, 0.68) |
| **SUD caseload** |  |  |
| Number of SUD clients in the past 30 days |  |  |
| *2 or less clients* | Reference |  |
| *3 to 6 clients* | 1.58 | (1.00, 2.48) |
| *13 to 7 clients* | 2.91 | (1.79, 4.70) |
| *14 or more clients* | 6.69 | (4.17, 10.73) |
| Treated a client with a covid-19 diagnosis | 1.46 | (1.06, 2.03) |
| Percentage of clients aged 40 or over | 1.00 | (0.99, 1.00) |
| Percent SUD/OUD clients with a CCI over 1 | 1.00 | (1.00, 1.01) |
| Percentage of clients prescribed benzodiazepines | 0.99 | (0.98, 1.00) |
| Percentage of clients accessing social assistance during follow-up | 1.02 | (1.02, 1.03) |
| Percentage of clients experienced an overdose in the past 12-months | 1.00 | (0.99, 1.01) |

**Table A13.** Logistic regression results for probability of PSS uptake when ending the calendar month on the 15^th^ of each month.

|  | Odds ratio | 95% CI |
| --- | --- | --- |
| Study month | 1.03 | (1.01, 1.04) |
| **Network characteristics** |  |  |
| Percentage of connected physicians that prescribed PSS prior months |  |  |
| *None* | Reference |  |
| *10% or less* | 1.62 | (1.24, 2.11) |
| *>10% to 20%* | 2.34 | (1.87, 2.92) |
| *>20%* | 5.08 | (4.16, 6.19) |
| Percentage of connected physicians that are also connected | 0.99 | (0.99, 1.00) |
| Number of additional physicians their average clients saw | 1.60 | (1.46, 1.76) |
| **Practice characteristics** |  |  |
| Primary HA practice |  |  |
| *Interior* | Reference |  |
| *Fraser* | 0.78 | (0.63, 0.97) |
| *Vancouver Coastal* | 1.28 | (1.05, 1.57) |
| *Vancouver Island* | 1.17 | (0.95, 1.46) |
| *Northern* | 1.30 | (1.00, 1.71) |
| *Unknown* | 0.94 | (0.49, 1.83) |
| Physician speciality |  |  |
| *General practice* | Reference |  |
| *Nurse practitioner (non-physician)* | 1.44 | (1.13, 1.82) |
| *Psychiatry* | 1.11 | (0.87, 1.41) |
| *Known but other specialties* | 1.57 | (1.14, 2.18) |
| *Unknown (physician)* | 0.46 | (0.34, 0.62) |
| OAT prescriber | 2.93 | (2.48, 3.45) |
| **SUD prescribing history** |  |  |
| Years since first billing record for a client with SUD/OUD |  |  |
| *<4.5 years* | Reference |  |
| *4.5 to <10.9 years* | 0.86 | (0.73, 1.02) |
| *10.9 to <22.4 years* | 0.78 | (0.65, 0.93) |
| *≥ 22.4 years* | 0.66 | (0.55, 0.8) |
| **SUD caseload** |  |  |
| Number of SUD clients in the past 30 days |  |  |
| *2 or less clients* | Reference |  |
| *3 to 6 clients* | 1.64 | (1.17, 2.29) |
| *13 to 7 clients* | 2.23 | (1.57, 3.16) |
| *14 or more clients* | 5.74 | (4.13, 7.97) |
| Treated a client with a covid-19 diagnosis | 1.62 | (1.27, 2.07) |
| Percentage of clients aged 40 or over | 0.99 | (0.99, 1.00) |
| Percent SUD/OUD clients with a CCI over 1 | 0.99 | (0.99, 1.00) |
| Percentage of clients prescribed benzodiazepines | 0.99 | (0.99, 1.00) |
| Percentage of clients accessing social assistance during follow-up | 1.02 | (1.02, 1.02) |
| Percentage of clients experienced an overdose in the past 12-months | 1.00 | (0.99, 1.01) |

**References**

1. Ministry of Justice. Health Care Services Manual. In: Adult Custody Division Corrections Branch, editor. 2013.

2. British Columbia Centre on Substance Use. Risk Mitigation in the Context of Dual Public Health Emergencies: Update to Interim Clinical Guidance. 2022 [Available from: <https://www.bccsu.ca/wp-content/uploads/2022/02/Risk-Mitigation-Guidance-Update-February-2022.pdf>.

3. Government of Canada. Guidance for repeated PCR testing in individuals previously positive for COVID-19. 2020.

4. Piske M, Zhou H, Min JE, Hongdilokkul N, Pearce LA, Homayra F, et al. The cascade of care for opioid use disorder: a retrospective study in British Columbia, Canada. Addiction. 2020;115(8):1482-93.

5. Quan H, Sundararajan V, Halfon P, Fong A, Burnand B, Luthi JC, et al. Coding algorithms for defining comorbidities in ICD-9-CM and ICD-10 administrative data. Medical care. 2005;43(11):1130–9.

6. Degenhardt L, Randall D, Hall W, Law M, Butler T, Burns L. Mortality among clients of a state-wide opioid pharmacotherapy program over 20 years: risk factors and lives saved. Drug Alcohol Depend. 2009;105(1-2):9−15.
